# Effects of Implant–Abutment Connection Type and Inter-Implant Distance on Inter-Implant Bone Stress and Microgap: Three-Dimensional Finite Element Analysis

**DOI:** 10.3390/ma14092421

**Published:** 2021-05-06

**Authors:** Takashi Matsuoka, Tamaki Nakano, Satoshi Yamaguchi, Shinji Ono, Shota Watanabe, Takumi Sato, Hirofumi Yatani

**Affiliations:** 1 Department of Fixed Prosthodontics, Osaka University Graduate School of Dentistry, Osaka 565-0871, Japan; t-matsuoka@dent.osaka-u.ac.jp (T.M.); onoshin@dent.osaka-u.ac.jp (S.O.); swatanabe@dent.osaka-u.ac.jp (S.W.); takumi_sato@dent.osaka-u.ac.jp (T.S.); yatani@dent.osaka-u.ac.jp (H.Y.); 2 Department of Biomaterials Science, Osaka University Graduate School of Dentistry, Osaka 565-0871, Japan

**Keywords:** biomechanics, dental implants, CAD-CAM, finite element analysis

## Abstract

The attainment of a good aesthetic outcome in dental implant treatment requires inter-implant papilla reconstruction, which is very difficult to perform. Maintenance of the inter-implant bone is essential for maintenance of the inter-implant papilla. The aim of this study was to investigate the mechanical influences of the implant–abutment connection type and inter-implant distance on the inter-implant bone by using three-dimensional finite element analysis. Three computer-aided design models of two-piece implants were designed: external connection (EC), internal connection (IC), and conical connection (CC). In each model, two identical implants were placed with inter-implant distances of 3.0, 2.5, and 2.0 mm. The maximum principal stress and microgap were evaluated. The stress values of the inter-implant bone decreased in the following order: IC, EC, and CC. The microgap decreased in the following order: EC, IC, and CC. Regardless of the type of implant–abutment connection, the stress of the inter-implant bone increased as the inter-implant distance decreased. The microgap barely changed as the inter-implant distance decreased. A CC implant is a mechanically advantageous implant–abutment connection type for maintenance of the inter-implant bone. With an inter-implant distance of less than 3.0 mm, use of a CC implant might suppress absorption of the inter-implant bone.

## 1. Introduction

Dental implant treatment has recently become a popular option for restoration of missing teeth and human masticatory function [1]. However, bone resorption can reportedly occur around the implant neck after connecting the implant superstructure to the abutment, potentially resulting in recession of the soft tissue around the implant body [1,2,3]. Various factors have been reported as causes of bone resorption around the implant; two of these factors, overloading and microgap formation, are influenced by the type of implant–abutment connection [4]. Overloading may result in illness due to prosthetic or biological tolerance. Bone resorption around the implant is caused by microfracture of the bone when a certain amount of stress is applied to the bone [5]. In dental implant treatment, excessive occlusal force that is overloaded from the superstructure to the bone surrounding the implant through the implant–abutment connection is closely involved in bone resorption after the functional load is started [6]. However, the application of stress within a certain range to the bone does not result in bone resorption, and may cause bone expansion [5]. Nevertheless, no reports have described an increase in the bone mass around the implant in clinical practice, suggesting that excessive stress on the bone around the implants must be prevented.

Aesthetics are greatly impaired by recession of soft tissue accompanied by bone resorption, especially in the area of the maxillary anterior teeth. Although reconstruction of an inter-dental papilla is aesthetically important, such reconstruction between implants is considered more difficult than reconstruction of the inter-dental papilla between the implant and natural tooth or between the implant and pontic [7]. Therefore, alveolar bone resorption between implants must be suppressed to maintain the inter-dental papilla between implants.

Peri-implant bone resorption is a complicated phenomenon with various etiologies such as insufficient oral hygiene, lack of adhesive gingiva, overload on the bone, overheating, and bacterial infection due to microgaps between the implant body and abutment [8,9]. Among these, overload on the bone and microgap formation between the implant body and abutment are influenced by the particular implant–abutment connection type, which is one criterion that affects implant selection in clinical practice. As the implant–abutment connection is dynamically weak, it is necessary for microgaps between the implant body and abutment to resist bacterial penetration and to resist occlusal force, which is an important component related to bone resorption [4,10]. Various implant–abutment connection types are currently available, but no mechanical studies have indicated which design most effectively maintains the inter-implant bone.

Inter-implant bone resorption is also affected by implantation conditions such as the inter-implant distance. Inter-implant distances of less than 3.0 mm result in increased amounts of bone resorption [11,12]. However, accurate placement of implants still has the possibility of errors in the implant position and inter-implant distance [13]. No reports have described the mechanical effects of various inter-implant distances on inter-implant bone.

Finite element analysis (FEA) is a useful method for predicting the long-term prognosis of prosthetic dental treatment in an oral environment with simulated load conditions. FEA has been used to evaluate stress transmission from the implant to the bone [14]. Many previous studies analyzed stress using a three-dimensional scanner with analysis software to reproduce implant components that are small in size and complicated in form [15]. In such studies, the analysis model becomes complicated, and the calculation cost inevitably increases due to the enormous amount of data. Many stress analyses were performed for only one implant, and many analysis models did not include a superstructure.

Therefore, the present study used three-dimensional FEA to compare the mechanical effects of differences in the implant–abutment connection type and inter-implant distance on inter-implant bone in the maxillary anterior region. Stress evaluation of the implant component was also performed to elucidate the transmission of mechanical stress on the bone.

## 2. Materials and Methods

**Figure 1:**
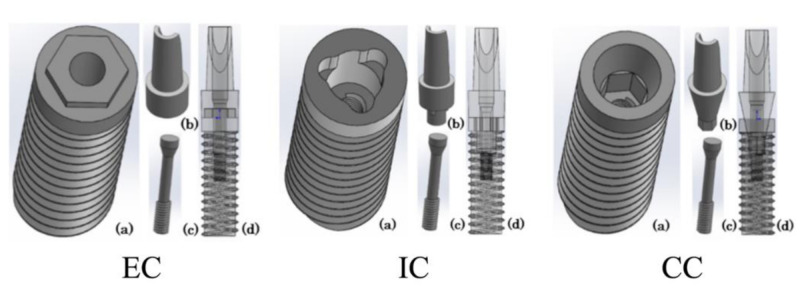
Three-dimensional computer-aided design models. (**a**) Implant body. (**b**) Abutment. (**c**) Abutment screw. (**d**) Model combining (**a**–**c**). EC: external connection; IC: internal connection; CC: conical connection.

**Figure 2:**
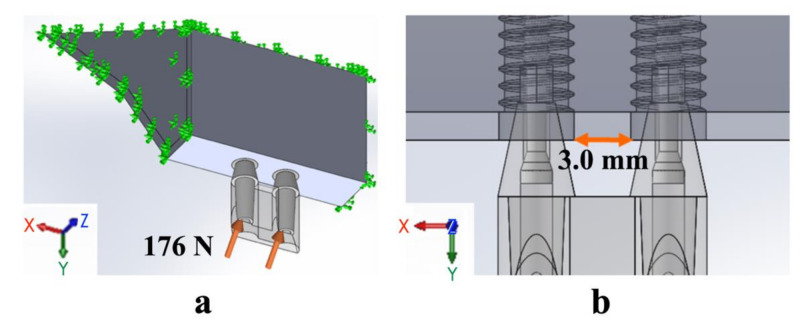
Implant and surrounding maxillary bone used for finite element analysis and modeling. (**a**) Overview of the conical connection (CC) model. (**b**) View of the CC model from the labial side. Two implants are implanted so that the inter-implant distance is 3.0 mm.

Three different computer-aided design (CAD) models of two-piece implants were designed using CAD software (SolidWorks 2013; Dassault Systèmes SolidWorks Corporation, Waltham, MA, USA): external connection (EC), internal connection (IC), and conical connection (CC) (Figure 1). The diameter × length of the implant body was 4 × 13 mm. The shape of the threads and abutment screw were the same in all models. The models also had the same shape of the implant body and abutment, excluding the part related to the connection. The implant body and abutment were connected by an abutment screw. A CAD model of an anterior maxillary bone with a 1.5-mm-thick cortical bone and cancellous bone was prepared. Two identical implants were placed at inter-implant distances of 3.0, 2.5, and 2.0 mm in the bone model, and a crown was connected to each abutment (Figure 2). The mechanical properties of the finite element models are shown in Table 1.

The “fixed bond” condition was set at the interface of the bone–implant and abutment–metal substructure by assuming osseointegration and cementation. The “contact” condition with friction coefficient (0.3) was set at the interfaces of the implant components for simulations of abutment micromovement. The bottom part and both sectional surfaces of the maxillary bone were fixed. A static load of 176 N was applied to each basal ridge surface of the metal substructure at a 45° oblique angle from the palatal side to the long axis of the implant (Figure 2a) [12]. The positive direction of the Z axis indicates the labial side, while the negative direction indicates the palatal side. The X axis and the Z axis indicate the mesial distal direction and the implant axial direction, respectively (Figure 2b). The element used for FEA was a tetrahedron, and the number of elements was determined by performing a convergence test based on the maximum principal stress. SolidWorks Simulation (Dassault Systèmes SolidWorks Corporation) was used for three-dimensional FEA.

The stress of the inter-implant bone and implant component and the microgap between the implant body and abutment were evaluated. The stress distribution was obtained by cutting the bone model with a plane passing through the center of the two implant bodies and evaluated when viewed from the buccal side at an angle of 45° with respect to the implant axis. The stress distribution of the implant component was evaluated in the same way as for the inter-implant bone. A microgap was evaluated as a gap at the implant–abutment interface when a load was applied to each model. The cross-section YZ-plane of the implant component in the buccopalatal direction was evaluated.

## 3. Results

**Figure 3:**
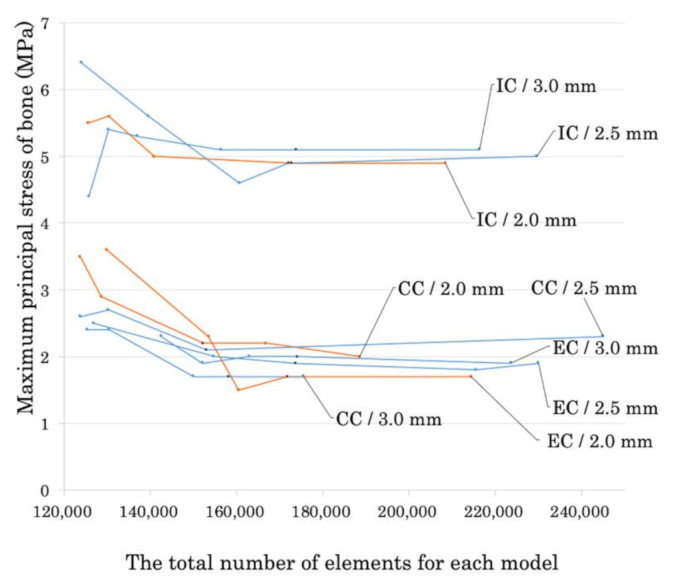
Convergence test results based on the maximum principal stress of bone. The black dots indicate the number of elements where the value of the maximum principal stress converged. EC: external connection; IC: internal connection; CC: conical connection. The mm values indicate the inter-implant distance for each model.

The results of the convergence study are shown in Figure 3, and the number of elements for each model are shown in Table 2.

**Figure 4:**
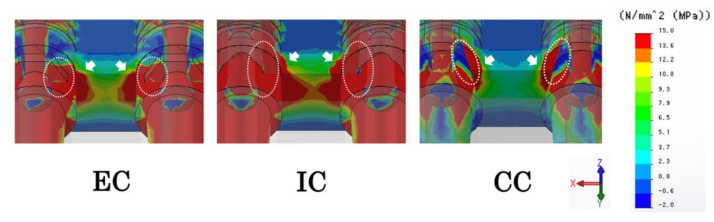
Maximum principal stress distribution of the bone and implant components at an inter-implant distance of 3.0 mm.

**Figure 5:**
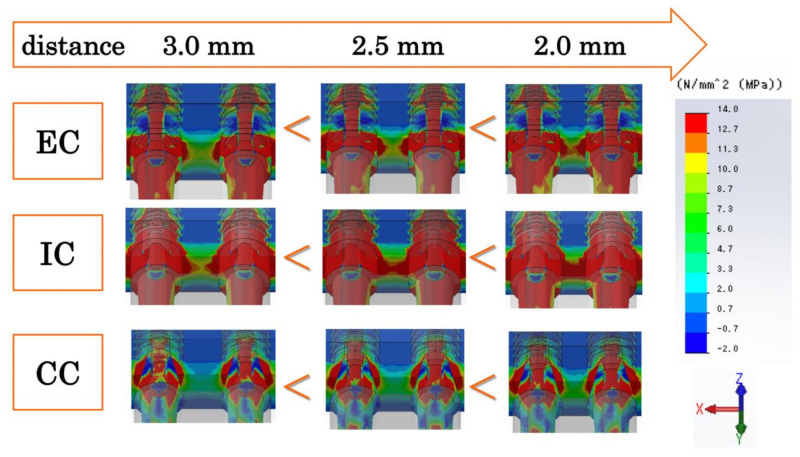
Maximum principal stress distribution of the bone and implant model at the three assessed inter-implant distances. The < and > signs indicate the relationships between models regarding the magnitude of the range of the stress distribution. EC: external connection; IC: internal connection; CC: conical connection.

At the inter-implant distance of 3.0 mm, the tensile stress in the mesial direction from the two implants overlaps the inter-implant bone (Figure 4). In the EC model, tensile stress concentrates toward the abutment side at the implant–abutment connection (white dotted line), and there is greater tensile stress in the inter-implant bone (white arrow) than in the CC model (Figure 4). In the IC model, tensile stress concentrates on the implant side in addition to the abutment side at the implant–abutment connection (white dotted line), tensile stress is applied from the apical part by the inter-implant bone (white arrow), and there is greater tensile stress than in the EC model (Figure 4). In the CC model, there is tensile stress on the implant side and compressive stress on the abutment side at the implant–abutment connection (white dotted line), and there is less tensile stress in the inter-implant bone (white arrow) than in the EC and IC models (Figure 4). The stress distribution range of the inter-implant bone decreased in the following order: IC, EC, CC (Figure 4 and Figure 5).

**Figure 6:**
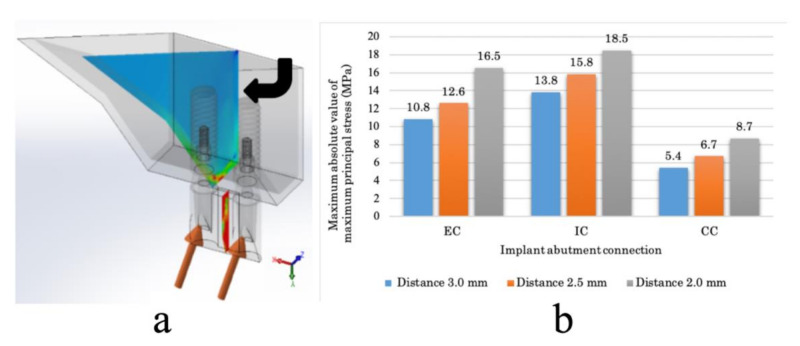
Stress value of the inter-implant bone. (**a**) Buccolingual plane in the midline of the inter-implant bone (black arrow). (**b**) Maximum value of the maximum principal stress of the inter-implant bone in each model. EC: external connection; IC: internal connection; CC: conical connection.

The stress distribution and stress value of the inter-implant bone increased as the inter-implant distance decreased in all models (Figure 5 and Figure 6). The CC model showed significantly lower stress values than the EC and IC models, regardless of the inter-implant distance.

**Figure 7:**
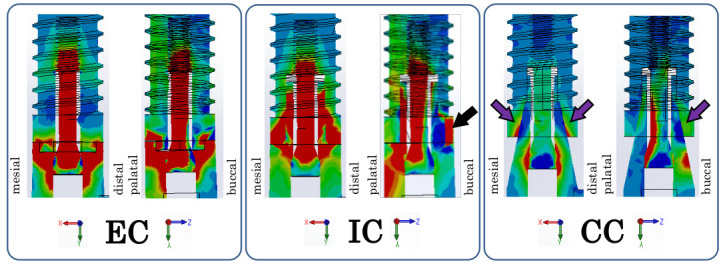
Maximum principal stress distribution of each implant components at inter-implant distance of 3.0 mm. Cross-sectional view of the implant component in the mesiodistal direction on the left side and the buccolingual direction on the right side.

Figure 7 shows the maximum principal cross-sectional stress distribution of the implant component in the mesiodistal and buccopalatal directions. In the EC model, the stress was concentrated on the abutment side of the implant–abutment connection. In the IC model, the stress was concentrated on both the implant and abutment sides of the connection. The black arrow indicates where the stress distribution is reversed on the buccal side of the interface between the abutment and the implant body. There was compressive stress on the implant side and tensile stress on the abutment side. In the CC model, there was tensile stress on the implant side and compressive stress on the abutment side of the connection, and the stress was dispersed around the connection. The purple arrow indicates where the stress distribution is reversed on the buccal side and mesiodistal side of the interface between the abutment and the implant body. There was compressive stress on the implant side and tensile stress on the abutment side.

**Figure 8:**
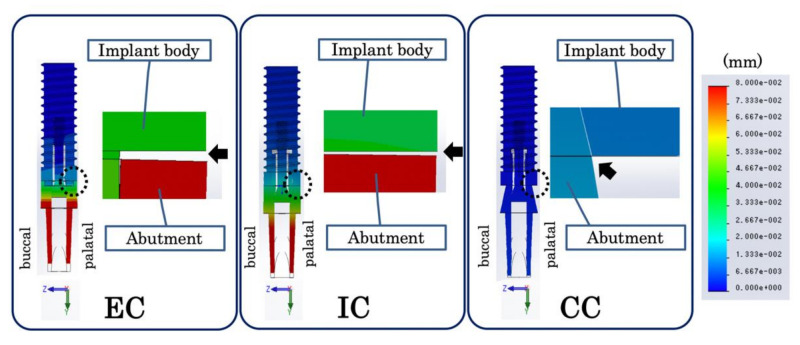
(**a**) Microgap measurement on a cross-sectional view of the implant component in the buccopalatal direction. The figure on the right is an enlargement of the region indicated by the dotted line. The black arrow indicates the microgap on the palate side of the interface between the implant body and the abutment. EC: external connection; IC: internal connection; CC: conical connection.

**Figure 9:**
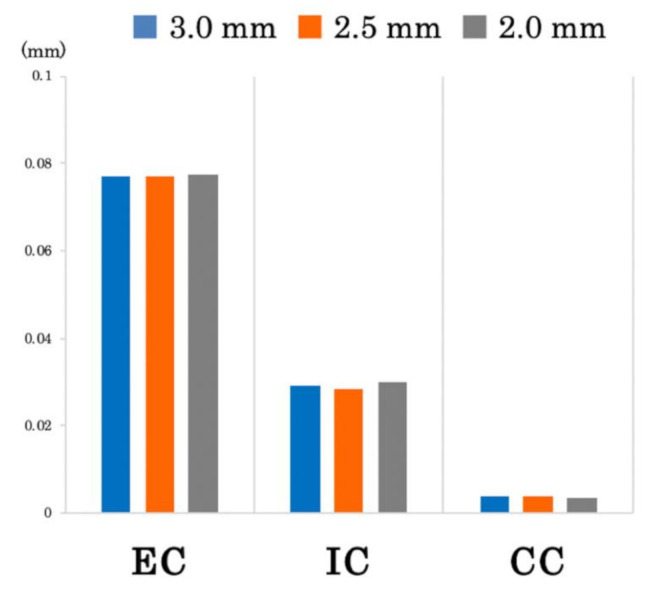
Size of the microgap between the abutment and the implant body in each model. EC: external connection; IC: internal connection; CC: conical connection.

At the same inter-implant distance, the size of the microgap decreased in the following order: EC, IC, CC (Figure 8). For all connection types, there was almost no change in the size of the microgap, even when the inter-implant distance changed (Figure 9). The CC model showed a much smaller microgap than the EC and IC models, regardless of the inter-implant distance.

## 4. Discussion

A microgap is a gap generated by micromovement of the abutment and implant body when a load is applied to the superstructure. Microgaps are a site of bacterial growth and cause bone resorption [11]. Although no microgap formation occurs in association with one-piece implants, two-piece implants are often selected to produce an aesthetically ideal superstructure; thus, microgaps need to be considered. As reducing the microgap may suppress bone resorption around the implant, various types of implant–abutment connections have become available.

Clinical studies, in vivo experiments, and in silico experiments using FEA have been performed to assess the influence of different implant–abutment connection types on the peri-implant bone of one implant. A recent clinical study used cone beam computed tomography to assess the three-dimensional temporal change in the labial alveolar bone, which cannot be evaluated on conventional dental X-ray images, and the results showed that a CC implant is advantageous for maintenance of the bone volume [18]. Similarly, an in vivo experiment showed that CC implants are advantageous for maintenance of the bone surrounding the implant [19]. In silico experiments have shown that the stress of the surrounding bone is low when using CC implants [14,15,20]. The microgap associated with CC implants is also small [15,21,22]. However, many in silico experiments were performed without accounting for crowns [15,21,22], and the abutment and abutment screw were united with the CC implant [14,15,20]; in many cases, there was no consistency among the models being compared. Moreover, few reports have described the influence of differences in the abutment connection type on the inter-implant bone of two adjacent implants. In clinical research, such assessments have only been performed using conventional dental X-ray imaging [16]. The artifacts that occur in cone beam computed tomography prevent the clear evaluation of the inter-implant bone in a small area of several millimeters. No in vivo experiments have considered differences in the abutment type, and only one type of abutment connection has been evaluated [12,17]. No in silico experiments have assessed the inter-implant bone between two adjacent implants. Therefore, there is a need for in vivo and in silico experiments on the inter-implant bone, which is difficult to assess in clinical research.

In the present study, the model creation and stress analysis were performed with one type of CAD software to markedly reduce the amount of data. The focus of the present study was the effect of differences in the implant–abutment connection type on the inter-implant bone. Therefore, the parts related to the connection (i.e., the implant body, abutment, and abutment screw) needed to precisely reproduce those in the actual structure, and they were separately designed and subsequently combined to produce the implant component model. All implant components other than the parts related to the connection were made in an identical manner for optimal consistency. Additionally, the analysis was performed with the crown on the abutment. To reduce the calculation cost, the analysis was performed using a precise implant model with the crown and no more than two implants, and comparative examination was possible when the experimental conditions were changed in small increments (0.5 mm).

The maximum occlusal force in the anterior teeth is reportedly 176 N [23]. In the present study, a static load of 176 N was therefore applied to each of the two implants. The maximum principal stress was selected as the evaluation criterion with which to evaluate the directionality in addition to the magnitude of the stress. Tensile stress occurs when the maximum principal stress value is positive, while compressive stress occurs when it is negative; thus, the directionality of the stress can be distinguished. The von Mises stress is often adopted as the evaluation criterion for stress [14]; however, unlike the maximum principal stress, it cannot be used to evaluate the directionality of stress.

In the present study, the tensile stress from the two implants overlapped on the inter-implant bone in the mesial direction (Figure 5 and Figure 6). Therefore, evaluating the stress distribution in the mesiodistal cross-section of the implant component enabled the attainment of information important for elucidating the stress transmission. The microgap was measured with reference to the palatal side, where the gap between the implant body and abutment was maximized. As the implant component deforms under loading, the size of the microgap at the implant–abutment interface will not be uniform. However, the bone surrounding the implant absorbs about 1 to 2 mm vertically or horizontally around the implant–abutment interface [11]. This is considered to be caused by the spread of bacteria throughout the interface when bacteria enter the microgap. In the present study, the load was applied from the palate side of the crown, and measuring the microgap on the palate side enables the determination of the size of the gap of the entire interface. Therefore, when evaluating the microgap, it is necessary to consider the stress distribution of the buccopalatal surface of the implant component.

Historically, to counteract the loosening of the abutment screw of the dental implant, it has been recommended to splint the superstructure of two adjacent implants [24]. Due to loosening of the abutment screw, the microgap may become large and cause bone resorption. Moreover, by connecting the crown, the stress is dispersed and effectively suppresses bone resorption [25]. Despite the fact that all analytical models in the present study involved a splinted crown, the microgap and stress of the inter-implant bone differed between models. These results suggest that the implant–abutment connection type is an important factor that indirectly affects bone resorption.

In the CC model, there was tensile stress on the implant side and compressive stress on the abutment side at the implant–abutment connection section in contact with the inter-implant bone (Figure 5 and Figure 6). In other words, reversal of the stress distribution occurred at the connection part, and stress cancellation resulted in a small amount of tensile stress on the inter-implant bone. In material dynamics, a substance deforms when subjected to external forces, resisting this deformation and generating internal stress [26]. Although the stress is uniformly distributed inside a simple shape, the distribution becomes uneven when the shape becomes complicated; large stress is generated in the uneven portion, and stress concentration occurs [26]. Unlike IC and EC implants, a CC implant has a unique shape without a concavo-convex structure in the part where the stress is most concentrated. In addition, a CC implant exhibits small micromovement of the abutment and implant body [15,21,22]. That is, as there is only a small amount of deformation of the constituent elements, stress concentration does not easily occur in any particular part. Additionally, on the buccal and mesiodistal sides of the interface between the abutment and implant body, there was tensile stress observed on the implant body side and compressive stress observed on the abutment side (Figure 8). This indicates that as the opposite stress that resists the sliding of the abutment acts on the interface, the micromovement of the abutment becomes smaller; this seems to explain the minimization of the microgap.

In the EC model, tensile stress concentration was observed near the abutment side of the implant–abutment connection in contact with the inter-implant bone (Figure 8). Thus, a larger tensile stress than that observed in the CC model was exerted on the inter-implant bone, without the cancellation of the stress at the connecting part that occurred in the CC model. The abutment of the EC was connected so that it rode on the implant body, stress was concentrated on the abutment side because of the uneven structure of the connection part on the abutment side, and the displacement of the abutment increased. Additionally, the microgap was maximized because the reverse stress distribution observed in the CC model was not observed in the EC model (Figure 8).

In the IC model, greater tensile stress than that in the EC model was concentrated on the implant–abutment connection, stress acted deep in the inter-implant bone, and tensile stress greater than that in the EC and CC models occurred (Figure 5 and Figure 6). As the concavo-convex structure of the connecting part was present on the implant body side and the abutment deeply entered the inside of the implant body, stress was concentrated on the implant body side in addition to the abutment side. Additionally, a reversed stress distribution that resisted sliding of the abutment seen in the CC model was observed only on the buccal side of the abutment–implant interface (Figure 8), resulting in a larger microgap than in the CC model.

The present study examined the mechanical influence of the inter-implant distance on the inter-implant bone. In terms of selectability by the operator, the inter-implant distance is as important as the implant–abutment connection. As the inter-implant distance decreased, the volume of the inter-implant bone decreased and the overlapping range of stress increased; thus, the stress of the inter-implant bone increased. In contrast, there was no significant change in the microgap as the inter-implant distance changed. This is because the distance from the loading point to the implant–abutment connection (fulcrum) barely changed. When implanting two adjacent EC implants in vivo, the horizontal bone resorption overlaps when the inter-implant distance is less than 3.0 mm, and the height of the alveolar bone and interdental papilla between the implants are lost [12]. The results of the present study support these findings from the viewpoint of stress. However, when CC implants are used in vivo, even when the inter-implant distance is 2.0 mm, there is a possibility of maintaining the inter-implant bone height equally, as in cases involving a 3.0-mm inter-implant distance [17]. In the present study, stress was lower than in the EC and IC models than the CC model, even when the inter-implant distance was small.

There was no significant change in the stress distribution patterns in the implant components in any of the implant–abutment connection types. In the CC model, there was no change in the stress distribution pattern, such as the stress dispersion within the implant component or stress distribution resisting slippage of the abutment, even when the inter-implant distance changed. Therefore, neither the stress nor the microgap was influenced by the embedding condition and became smaller than in the other connection types. In the clinical setting, the implantation position may be shifted, and the inter-implant distance may be smaller than the conventional distance of 3.0 mm. Even in such cases, the absorption of the inter-implant bone might be suppressed by using CC implants.

The analyses performed in the present study were linear and static. Nonlinear and dynamic analyses [27,28,29] may provide findings in an environment closer to actual clinical cases, likely allowing for more accurate evaluation of the effects of differences between the implant–abutment connection type and inter-implant distance.

## 5. Conclusions

The purpose of this study was to determine the implant–abutment connection type and implantation condition that are most mechanically advantageous for maintenance of the inter-implant bone when two implants are inserted consecutively in the aesthetic area. Stress analysis using three-dimensional FEA revealed the following:

1. When implanting two adjacent implants in the region of the maxillary anterior teeth, a CC implant is a mechanically advantageous implant–abutment connection type for maintenance of the inter-implant bone.
2. A small inter-implant distance is mechanically disadvantageous for maintenance of the inter-implant bone.
3. With an inter-implant distance of less than 3.0 mm, use of a CC implant might suppress absorption of the inter-implant bone.

## Figures and Tables

**Table 1:** Mechanical properties used for the three-dimensional finite element analysis model.

Component	Material	Elastic Modulus (MPa)	Poisson Ratio	References
**Cortical bone**	Cortical bone	13,000	0.3	[16]
**Cancellous bone**	Cancellous bone	1370	0.3	[16]
**Implant body**	Titanium	117,000	0.3	[16]
**abutment**				
**Abutment screw**	Titanium alloy	120,000	0.36	[17]
**Superstructure**	Gold alloy	96,600	0.35	[14]

**Table 2:** Number of elements of the analysis model.

Connection Type, Inter-Implant Distance	Number of Elements
External connection, 3.0 mm	162,963
External connection, 2.5 mm	173,526
External connection, 2.0 mm	171,671
Internal connection, 3.0 mm	156,325
Internal connection, 2.5 mm	172,679
Internal connection, 2.0 mm	171,898
Conical connection, 3.0 mm	155,243
Conical connection, 2.5 mm	152,925
Conical connection, 2.0 mm	152,115

## Data Availability

Data sharing not applicable.
